# Supplementary material for: Ground beetle assemblages inhabiting various age classes of Norway spruce stands in north-eastern Poland
Source: PeerJ. 2023 Dec 1;11:e16502. doi: 10.7717/peerj.16502 (PMC10695117; doi:10.7717/peerj.16502)
Supplement: Supplemental Information 2 [file peerj-11-16502-s002.docx]

The results of statistical analyses are presented in the Table 3 and on figures 3a, 3b and 3c. Below we reported details of statistic we used in the manuscript.

|  | df | Wald’s statistic | p value |
| --- | --- | --- | --- |
| Individuals | 2 | 45.646 | 0.000000 |
| Species | 2 | 84.7538 | 0.00 |
| MIB | 2 | 173.1 | 0.00 |

Homogenous groups

| Nr podkl. | Bonferroni’s test; **Individuals**, Homogenous groups, alfa = ,05000 Error: MS Effect = 17,363, df = 657,00 | | | |
| --- | --- | --- | --- | --- |
|  | \| The age of the forest \| \| --- \| | \| Individuals Medium \| \| --- \| | \| 1 \| \| --- \| | \| 2 \| \| --- \| |
| \| 1 \| \| --- \| | C | 3,895455 |  | **** |
| \| 2 \| \| --- \| | B | 5,100000 | **** |  |
| \| 3 \| \| --- \| | A | 5,118182 | **** |  |

| Nr podkl. | Bonferroni’s test; **Species**, Homogenous groups, alfa = ,05000 Error: MS Effect = 2,1012, df = 657,00 | | | | |
| --- | --- | --- | --- | --- | --- |
|  | \| The age of the forest \| \| --- \| | \| Individuals Medium \| \| --- \| | \| 1 \| \| --- \| | \| 2 \| \| --- \| | \| 3 \| \| --- \| |
| \| 1 \| \| --- \| | C | 1,495455 | **** |  |  |
| \| 2 \| \| --- \| | B | 2,431818 |  | **** |  |
| \| 3 \| \| --- \| | A | 2,781818 |  |  | **** |

| Nr podkl. | Bonferroni’s test; **MIB**, Homogenous groups, alfa = ,05000 Error: MS Effect = 7441,2, df = 57,000 | | | |
| --- | --- | --- | --- | --- |
|  | \| Age \| \| --- \| | \| MIB Medium \| \| --- \| | \| 1 \| \| --- \| | \| 2 \| \| --- \| |
| \| 3 \| \| --- \| | A | 273,8185 | **** |  |
| \| 2 \| \| --- \| | B | 286,6709 | **** | **** |
| \| 1 \| \| --- \| | C | 341,8430 |  | **** |
